# Assessing Saudi medical students learning approach using the revised two-factor study process questionnaire

**DOI:** 10.5116/ijme.5974.7a06

**Published:** 2017-08-17

**Authors:** Shaffi Ahamed Shaik, Ahmed Almarzuqi, Rakan Almogheer, Omar Alharbi, Abdulaziz Jalal, Majed Alorainy

**Affiliations:** 1 Department of Family and Community Medicine, College of Medicine, King Saud University, Riyadh, Saudi Arabia; 2 College of Medicine, King Saudi University, Riyadh, Saudi Arabia

**Keywords:** Study process, R-SPQ2F questionnaire, validity, Saudi medical students

## Abstract

**Objectives:**

To assess learning approaches of 1st, 2nd, and 3rd-year medical students by using revised two-factor study process questionnaire, and to assess reliability and validity of the questionnaire.

**Methods:**

This cross-sectional study was conducted at the College of Medicine, Riyadh, Saudi Arabia in 2014. The revised two-factor study process questionnaire (R-SPQ-2F) was completed by 610 medical students of both genders, from foundation (first year), central nervous system (second year), medicine and surgery (third year) courses. The study process was evaluated by computing mean scores of two research study approaches (deep & surface) using student’s t-test and one-way analysis of variance. The internal consistency and construct validity of the questionnaire were assessed using Cronbach’s α and factor analysis.

**Results:**

The mean score of deep approach was significantly higher than the surface approach among participants(t_(770)_=7.83, p= 0.000) for the four courses. The mean scores of deep approach were significantly higher among participants with higher grade point average (F_(2,768)_=13.31, p=0.001) along with more number of study hours by participants (F_(2,768)_=20.08, p=0.001). The Cronbach’s α-values of items at 0.70 indicate the good internal consistency of questionnaire used. Factor analysis confirms two factors (deep and surface approaches) of R-SPQ-2F.

**Conclusions:**

The deep approach to learning was the primary approach among 1st, 2nd and 3rd-year King Saud University medical students. This study confirms reliability and validity of the revised two-factor study process questionnaire. Medical educators could use the results of such studies to make required changes in the curriculum.

## Introduction

The learning approach and study methods are the bi-fold processes by which a student obtains, understands, and retains knowledge to perform better in examinations. The learning approach of students has always been a concern for teachers and medical education experts. The learning approach is linked with the nature of the relationship between student, context and task.^1^ Learning style varies from individual to individual according to an assigned task; even it influences a person's learning behavior in groups, solving the problems and interacting with educators. However, learning styles are stable traits that affect the learner's information processing and his cognition regarding attention, perception and thinking.^2^ In the field of medicine, medical students entail skill and competence in multiple disciplines and need to grab the wide range of acquired knowledge in the short period.^3^ As per Sadler-smith, learning style is defined as the distinctive and habitual manner of acquiring knowledge, skills and attitude through study or experience.^4^ Numerous methods of the learning styles are available in the literature, and each method offers the categorically different view of learning style preferences. These approaches to learning include Visual, Aural, Read/write, and Kinesthetic (VARK) styles,^5^ Concrete experience, Reflective Observation, Abstract Conceptualization and Active experimentation.^5^^,^^6^ There are many articles available in the literature that elaborates surface and deep learning approaches among undergraduate students. Students with surface approach quietly accept information and memorize it as isolated and unrelated facts. Students who adopt a deep approach to learning have an intrinsic motivation to study a subject area.^7^ The concept of surface and deep learning approaches is in one of the key features for the improvement of a self-directed learner. The coexistence can occur between the surface and deep learning approaches. Contingent factors will promote the learning style of the student.^8^ Gibbs and colleagues^9^ describes factors that contribute to deep learning as an integrated curriculum, match between the assessment and objectives, intrinsic motivation, and a learner-centered educational environment. Medical teaching is an ongoing process for students and teachers to keep them up-to-date.^10^ Biggs and colleagues^11^ have developed a revised two factors study purpose questionnaire (R-SPQ-2F) which can be used to assess the learning approaches of students. The College of Medicine, King Saud University (KSU), is one of the oldest medical colleges in the region that introduced block curriculum teaching method in 2010.  This new curriculum was designed with high priority for student self–learning when compared to an earlier traditional (lecture method) curriculum. A 5-year curriculum for undergraduate medical students was designed as in such a way that during the first two years, students deal with the whole comprehensive subjects or blocks for the body systems. The middle year of the course (i.e., 3rd year), medical students will be involved in medicine, surgery and clinical practice after completion of the course for basic medical sciences. In the 4th year, students will be trained for clinical specialities' such as ENT, Orthopedics, Ophthalmology, Anesthesia, Obstetrics and Gynecology. While in the final year, students will complete the practice in medicine, surgery and paediatrics.  With the change of curriculum in this college, we are interested to know the learning approaches of our undergraduate medical students. Therefore, this study was designed to assess the learning approaches of 1st, 2nd and 3rd-year medical students by using R-SPQ-2F questionnaire and also to evaluate the reliability and validity of this questionnaire.

## Methods

### Study design and setting

For this cross-sectional study, we selected 622 participants from College of Medicine, King Saud University (KSU). Selected participants were both male and female undergraduates pursuing medicine starting from 1st year to 3rd year of the course. The selected participants' curriculum blocks were the foundation, CNS, medicine, surgery and obstetrics and gynaecology. Ethical approval for this study was granted by Institutional Review Board of the college of medicine, King Saud University. All the participants signed the consent form and were explained about the importance of this study.

### Study participants

Out of initially selected 622 participants, only 610 (92.2%) showed interest and signed the consent form, and only those participants were included in this study who signed the informed consent. Among the selected participants, 221 were included in foundation block, 229 for CNS block and remaining 160 participants were accounted twice for Medicine and Surgery courses. For analysis purpose, the sample size was considered as 770. Out of 610 participants, 370(60.6%) were male.

### Instrument and procedures

Data were collected using R-SPQ-2F questionnaire which was developed by Biggs colleagues11. The questionnaire measures the deep and surface learning approaches. Each approach consists of 10 items and all together will be 20 items. A 5-point Likert scale was used to evaluate both the approaches (1= never or only rarely true of me & 5= always or almost always true to me).  The outcome of R-SPQ-2F was determined as the learning approaches whether it was a deep approach (Sum of deep motive and deep strategic) or surface approach (Sum of surface motive and surface strategic). Also, data of participant's gender, place of living, place of studying, Grade point average (GPA), some study hours on normal days and number of study hours during examinations were collected. Data collection was carried out during 14-20 weeks of the first semester and between 1st to 3rd weeks of the second semester of the academic year.

### Data analysis

Data were analysed using SPSS software, version 21.0. Descriptive statistics (mean & standard deviation) were used to describe the quantitative outcome variables. Student's t-test for the single sample was used to compare the mean score difference of deep and surface approaches. Student’s t-test for independent samples and one-way analysis of variance followed by Turkey's multiple range test was used to compare the mean values of quantitative outcome variable about the categorical study variable of two and more than two categories. Internal consistency of R-SPQ-2F was assessed using Cronbach’s alpha; convergent validity was evaluated using Karl Pearson’s correlation coefficient among the items, subscale scores and total scores.  The validity of R-SPQ-2F was determined by using factor analysis in which the correlation matrix, Kaiser-Meyer-Olkin (KMO) measurement of sampling adequacy and Bartlett’s test of sphericity were used to assess the factorability of the 20 items.  Factor structure was restricted to two factors in the factor extraction process by using a principal component method. The proportion of variance explained by each of the factors was assessed through Eigen-values. Varimax rotation was used to obtain the rotated factors. A p-value of ≤ 0.05 and 95% confidence intervals was used to report the statistical significance and precision of results.

## Results

Of the 610 participants, a higher GPA (4.5 to 5) was held by 378(62%); 172 (28%) had 4- 4.49; the remaining 61(10%) participants had 3-3.99 GPA. The comparison of mean scores of deep and surface approaches for each of the four courses shows highly statistically significant difference in the mean scores in which the mean scores of deep approach are significantly higher than the mean scores of surface approach for all the four courses of the curriculum. This suggests that most of the participants during these four courses are adopting the deep approach (Table 1).

The comparison of the mean score of deep approach about gender, place of living, GPA, place of study, duration of daily study hours and duration of study hours during exams shows, statistically significant difference in the mean score for GPA categories and duration of daily study hours of participants. The deep approach learning approach mean score is significantly higher in the participants of GPA of 4-4.5 and 4.5 -5.0 when compared with participants of GPA with less than 4 (F_(__2,768)_ =13.31, p=0.001). Also, the mean score is significantly higher in the participants who spent 3- 5 hours and 5 to 10 hours during non-exam days when compared with participants who spent only 2 hours for their studies (F_(2,768)_ =20.08, p=0.001), see  Table 2.

The internal consistency reliability of 20 items, two main factors (deep approach & surface approach) and its four sub scales (deep motive, deep strategy, surface motive and surface strategy) was assessed by calculating Cronbach's α. As we can see from Table 3, the average measure of Cronbach's α value of all the 20 items under the two factors, for the four types of curriculum and the whole group ranged from 0.673 to 0.711. For 10-items, Deep study approach is   0.703 to 0.775 whereas for Surface study approach is 0.730 to 0.763.  The values of Cronbach's alpha are close to the acceptable level of 0.70 which suggests a satisfactory estimate of the reliability of the questionnaire (see Table 3).

The correlation among the 20 items of an Instrument showed statistically significant correlation. The KMO measures the sampling adequacy which should be greater than 0.5 for a satisfactory factor analysis to proceed. The data show that KMO measure of 0.808 for all subjects, and for the participants of 4 curriculums, KMO values are: 0.743, 0.741.0.744 and 0.746 and Bartlett's tests of sphericity are significant (p=0.000). This indicates that the correlation matrix is not an identity matrix. The analysis of factors extraction along with their Eigen values, the percent of variance attributable to each factor, and the cumulative variance of the factors show that first factor accounts for 36.43% of the variance, the second factor accounts for 27.70% of the variance, with a cumulative variance of 64.13% for all subjects. A similar pattern was observed for the other four groups of the curriculum. The loadings of the 20 items on the two factors were extracted for all the participants and the participants of 4 group curriculum. The higher the absolute value of the loading, the more the factor contributes to the variable. The loading indicates that two factors have contributed to each of its ten items. The two factors are Deep approach with its 10-items (1, 2, 5, 6, 9, 10,13,14,17 & 18) and Surface approach with its 10-items (3, 4, 7, 8, 11, 12, 15, 16, 19 & 20, see Table 4).

**Table 1:** Comparison of mean scores of Deep Approach (DA) and Surface Approach (SA) learning methods of students during the curriculum course of foundation block, CNS block, Medicine and Surgery courses

Curriculum	DA	SA	Diff.	*t*	p-value	95% CI difference
Foundation	20.6(5.0)	17.5(4.8)	3.1	6.49	0.0001	(2.15,4,03)
CNS	19.5(4.8)	17.6(5.1)	1.9	4.04	0.0001	(0.94,2.72)
Medicine	19.3(5.2)	17.6(4.8)	1.7	2.85	0.005	(0.49,2.74)
Surgery	19.1(5.2)	17.9(5.1)	1.2	2.00	0.047	(0.01,2.35)
All Subjects	19.7(5.1)	17.7(5.0)	2.0	7.83	0.001	(1.51,2.52)

**Table 2:** Comparison of mean values of Deep Approach method of learning in relation to the study variables (N=770)

Variables				Deep approach Mean (SD)		t / F test		p-value	
	Gender							
		Male		19.56(4.9)		-0.934		0.350
		Female		19.91(5.3)				
	Place of living							
		Single		19.62(4.8)		-0.144		0.886
		With family and friends		19.68(5.1)				
	GPA							
		3-3.99		17.48(4.9)		13.31		0.001^*^
		4-4.49		19.32(4.4)				
		4.5-5		20.32(5.3)				
	Place of studying							
		With friends		20.19(5.6)		0.791		0.499
		Library		20.35(4.8)				
		Alone at home		19.63(5.0)				
		More than one place		19.29(5.2)				
	Study hours in a day							
		2 hours		18.62(4.6)		20.08		0.001^*^
		3-5 hours		20.67(5.3)				
		5-10 hours		22.0(5.2)				
	Study hours during exam							
		<=5 hours		19.19(4.8)		-1.54		0.123
		> 5 hours		19.85(5.2)				

## Discussion

In all the medical schools, much attention is given to the periodical development of curricular content, the scheduling of teaching and the conducting of examinations. But little attention is given to assess the effect of these activities on the learning process of the students. The aim of this study was to assess the learning approaches among KSU medical students and to examine the validity and reliability of the R-SPQ-2F questionnaire. The results show that the deep approach scores of the study participants are higher than the surface approach scores. This suggests that participants in all three years with different subjects (foundation block, CNS block, medicine and surgery) in our medical college preferred the deep approach to learning as compared to the surface approach. The comparison of these study findings with other studies may be difficult due to the use of different instruments to evaluate the learning approaches of students (medical and non-medical) in various settings. But most of the results also emphasize on the deep approach to learning.^12^^-^^14^ A study by Samarakoon and colleagues^10^ that included the first and final year of medical undergraduate, postgraduate and trainee students observed that the learning approach was strategic with the VARK and ASSIST (Approaches to study skills Inventory for students) questionnaires.

**Table 3:** Internal consistency (Cronbach's Alpha) of revised two-factor approach Instrument for all items, 10-items of DA and 10-items of SA across the four groups (foundation block, CNS block, medicine and surgery) and all subjects

Type of curriculum		Cronbach's alpha (95% CI)	
	20-items	10 items-(DA)	10 items-(SA)
Foundation block subjects	0.673(0.61,0.73)	0.706(0.64,0.76)	0.738(0.68,0.78)
CNS block subjects	0.713(0.66,0.76)	0.703(0.64,0.76)	0.763(0.71,0.80)
Medicine course subjects	0.704(0.63,0.77)	0.767(0.71,0.82)	0.730(0.66,0.79)
Surgery course subjects	0.711(0.64,0.77)	0.775(0.72,0.82)	0.755(0.69,0.81)
All subjects	0.699(0.67,0.73)	0.737(0.71,0.76)	0.746(0.72,0.77)

Another study by Ward et al^15^ using ASSIST questionnaire confirms deep approaches provide good results in their medical education. The mean scores of deep approaches by medical students were significantly higher in the study reported by Emilla et al^16^ by using SPQ (study process questionnaire).  In another study among Ghanaian medical students which had used R-SPQ-2F questionnaire also observed that deep approach was the most dominant learning among the students, which goes in line with our findings.^7^ By using ASSIST questionnaire, Cebeci and colleagues^17^ have found that medical students preferred deep and strategic approach rather than surface approach.  Similarly, Ravi and colleagues^18^ in their study had reported that second-year medical students had used deep and strategic learning styles. In contrast, the majority of first-year medical students of Erciyes University, Turkey were found to be using multimodal learning style which was assessed by using VARK questionnaire.^19^ The preference of deep approach of learning in our study participants is consistent with the study findings by Hilliard^20^ which demonstrates that medical students who adopted deep approach had higher scores when compared with those following surface approach for their learning. Results of this study also show that participants who had higher GPA (≥ 4.0) and those who spent more number of study hours (≥ 5 hours)  had significantly higher mean deep approach score than the other participants with less GPA and who spend a lower number of study hours. Similar to our findings, Lynch and colleagues^6^ in their study observed that academic performance is influenced by the learning styles among third medical students, but Wilkison and colleagues^21^ concluded that overall performance of first-year medical and dental students is not influenced by learning style. Another study among 1st-year medical students by Davies and colleagues^22^ found a significant impact of learning styles on their academic performance. Also, a positive correlation was observed between the learning approaches and the academic performance among the 3rd to 5th-year medical students.^20^ Like other studies, our findings also indicate that participants were achieving higher GPA (academic performance) by following the deep approach to their learning.

**Table 4:** Factor loadings of two factors Instrument of study approaches (Deep and Surface) across the four groups (foundation block, CNS block, medicine and surgery) and all study subjects

R-SPQ-2F and its items	Foundation Block		CNS Block		Medicine		Surgery		All study subjects	
	DA	SA	DA	SA	DA	SA	DA	SA	DA	SA
1	382	-.168	.526	-.174	.514	.042	.550	.033	.473	-.084
2	.444	-.130	.535	-.161	.594	-.044	.627	-.053	.535	-.125
3	.026	.548	-.016	.647	-.024	.597	-.120	.598	-.043	.598
4	-.155	.338	-.084	.476	.009	.504	-.020	.498	-.055	.436
5	.503	-.140	.392	.036	.629	.052	.618	.061	.523	-.018
6	.648	.007	.585	-.015	.615	-.285	.670	-.196	.631	-.109
7	.081	.542	-.101	.540	-.019	.487	.046	.471	.000	.525
8	.189	.437	.264	.485	.363	.379	.373	.488	.294	.441
9	.511	.078	.624	.068	.565	.023	.545	.147	.565	.079
10	.385	.142	.399	.014	.627	-.029	.637	-.001	.514	.032
11	.129	.654	.262	.381	.314	.445	.211	.531	.229	.515
12	-.271	.570	.156	.650	.047	.720	.030	.728	-.012	.668
13	.617	-.173	.611	-.111	.697	-.159	.636	-.160	.640	-.150
14	.622	.077	.514	.057	.547	-.002	.562	-.004	.567	.035
15	-.207	.585	-.050	.622	-.126	.423	-.173	.464	-.116	.549
16	-.028	.509	-.094	.576	-.146	.703	-.158	.724	-.108	.618
17	.514	.057	.429	.252	.275	-.016	.348	-.059	.423	.083
18	.548	.205	.594	.103	.489	.022	.446	-.049	.535	.098
19	.012	.638	.007	.605	-.067	.513	-.120	.486	-.037	.575
20	.071	.592	.005	.609	-.007	.587	-.023	.562	.021	.585

DA=Deep Approach (1, 2, 5, 6, 9, 10, 13, 14, 17, 18)

SA=Surface Approach (3, 4, 7, 8, 11, 12, 15, 16, 19, 20)

Our study results revealed the reliability and validity with R-SPQ-2F questionnaire and showed positive levels of internal consistency for these two factors (0.737 for deep approach and 0.746 for surface approach). The total variance explained by the two factors of R-SPQ-2F was 64.13%. Our analysis of construct validity (i.e., factor analysis) identified deep, and surface approaches in R-SPQ-2F across the four curriculum groups of our study participants. A variance of 49.80% and 33.57% for two factors (deep and surface) was found among the Ghanaian medical students.^7^ Similar results of validity and internal consistency of Arabic version of this questionnaire were reported in another study in a sample of 85 high school graduates in KSA.^8^^,^^16^ Another study which was carried out among non-medical students in Malaysia showed evidence of good reliability and validity of this questionnaire.^23^ High internal consistency and good validity of our study results confirm the application of this questionnaire to measure the method of learning approach medical students across other medical schools in KSA. Rahman and colleagues^24^ also recommended using this questionnaire to measure the learning approaches of university students, by obtaining the high Cronbach alpha values and valid two-factor structure in university students of Malaysia.

The strength of this current study was big sample size and high response from the study participants. The limitation of this study is of participant’s recall bias in responding to 20 items of a study process questionnaire used in this study.

## Conclusions

Learning approach among the participants of our study is significantly inclined towards deep approach rather than surface approach. The deep approach to learning in our participants could be due to the component of self-learning by students in the curriculum structure of our college. Future studies could be carried out using another validated questionnaire to further confirm the observed learning approach (deep) of our study participants and to know any changes in learning approaches of medical students from time to time. This will enable the medical education experts to carry out the appropriate changes in the curriculum. Also, this study confirms the internal consistency and constructs validity of the R-SPQ-2F questionnaire. 

### Acknowledgments

The authors extend their appreciations to the College of Medicine Research Centre, Deanship of Scientific Research, King Saud University, Riyadh, Kingdom of Saudi Arabia for financial support. We would like to thank students for their participation in this research. We would also like to thank male and female group leaders who helped us during data collection.

### Conflict of Interest

The authors declare that they have no conflict of interest.
